# Tale of a “Non-interacting” Additive in a Lithium-Ion Electrolyte: Effect on Ionic Speciation and Electrochemical Properties

**DOI:** 10.1021/acs.jpcc.1c09193

**Published:** 2022-01-24

**Authors:** Jeramie C. Rushing, Callie M. Stern, Noémie Elgrishi, Daniel G. Kuroda

**Affiliations:** Department of Chemistry, Louisiana State University, Baton Rouge, Louisiana 70803, United States

## Abstract

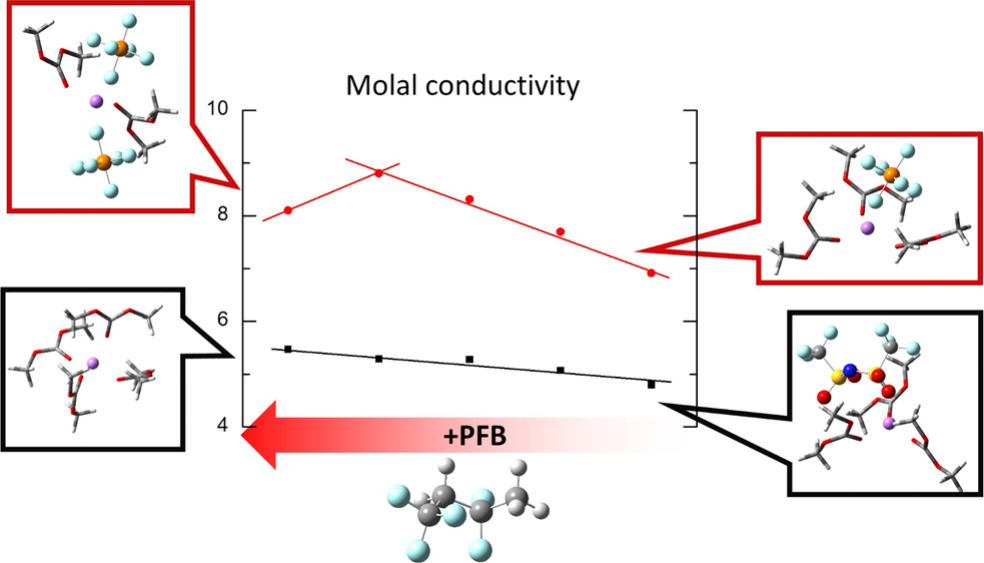

New lithium electrolytes compatible with high energy density cells are critical for lithium metal battery applications, but dendrite formation associated with the use of dilute organic electrolytes complicates their realization. High-concentration electrolytes mitigate some of the issues of the electrolytes but introduce additional problems, such as low conductivity and high cost. Hence, pseudo-concentrated electrolytes, wherein a co-solvent is added to a dilute electrolyte, have been presented as a possible alternative to both dilute and concentrated electrolytes. However, the effect that the co-solvent has on the electrolyte properties at both macroscopic and microscopic levels is unknown. Here, a study of the structure and electrochemical properties of two electrolytes as a function of co-solvent concentration is presented using an array of spectroscopies (FTIR, ATR–FTIR, and nuclear magnetic resonance) and computational methods (density functional theory calculations). The chosen electrolytes comprised two different lithium salts (LiPF_6_ and LiTFSI) in a mixture of dimethyl carbonate (DMC) with 1,1,1,3,3-pentafluorobutane (PFB) as the co-solvent. Our results show that in the case of the LiPF_6_/DMC electrolyte, the addition of a co-solvent (PFB) with a larger dielectric constant results in the strengthening of the lithium–anion interaction and the formation of aggregate species since PFB does not interact with the anion. Conversely, in the LiTFSI/DMC electrolyte, the co-solvent appears to interact with the anion via hydrogen bonds, which leads to the dissociation of contact ion pairs. The change in ionic speciation of the electrolytes upon addition of PFB provides a reasonable framework to explain the different trends in both the bulk and interfacial macroscopic properties, such as conductivity, viscosity, and electrochemical stability. Overall, our findings demonstrate that the interactions between the anion and the co-solvent must be taken into consideration when adding a co-solvent because they play a major role in determining the final electrolyte properties.

## Introduction

Lithium-ion batteries have become the dominant technology in portable energy storage due to their low weight, cost efficiency, and relatively high energy density since their introduction in 1991.^1^ The current commercial lithium-ion batteries, widely used in portable electronics, are based on a graphite anode, which lacks sufficient energy density to support more energy demanding applications, such as electric vehicles and power grid storage.^2−4^ To this end, lithium metal has the potential of becoming an anode material due to its exceptionally high theoretical specific capacity (3860 mA h g^–1^) and low negative electrochemical potential (−3.040 V vs SHE).^5^ However, an anode consisting of metallic lithium has inherent problems including dendrite formation (short-circuiting) and unmitigated growth of the solid electrolyte interface (decreased cycling efficiency and increased resistance).^6−8^ To solve these issues, highly concentrated lithium salt electrolytes have been proposed. These highly concentrated electrolytes not only inhibit dendrite formation but also have other desirable properties, such as enhanced electrochemical stability, reduced corrosivity of the aluminum current collector, and the formation of a more stable solid electrolyte interface, the so-called SEI.^9−11^ However, highly concentrated electrolytes are not free of problems since they have high viscosity, low ionic conductivity, and high cost, which hamper their widespread adoption.^12−15^ One proposed alternative to suppress dendrite formation and reduce the cost of the highly concentrated electrolyte is the introduction of a “co-solvent”. An essential characteristic of the co-solvent should be to have minimum interactions with the electrolyte components. In other words, the co-solvent is expected to not directly interact with the lithium ion or its counter ion when added to the electrolyte. Previous works with co-solvents include the use of dioxolane, water, acetonitrile, propylene carbonate, diethyl carbonate, toluene, hydrofluoroethers, dichloromethane, hexafluoroisopropyl methyl ether, and 1,1,2,2-tetrafluoroethyl-2,2,3,3-tetrafluoropropylether as co-solvents to battery electrolytes.^16−24^ It has been observed that in some cases, the addition of co-solvents to highly concentrated electrolytes does not result in the total disruption of a rigid three-dimensional structure and in turn creates pockets of a highly concentrated electrolyte with increased mobility.^19,22^ For these reasons, these diluted electrolytes have been described as pseudo-concentrated electrolytes. Pseudo-concentrated electrolytes have particularly interesting properties such as enhanced ionic conductivity, lower viscosity, and lower cost when compared to highly concentrated electrolytes.^25^ In addition, the possibility of using these electrolytes for high-voltage lithium-ion batteries has been recently shown.^21,26^

Highly halogenated co-solvents have been used to create pseudo-concentrated electrolytes since they are expected to lower the viscosity of the system without modifying the pre-existing interactions among the components of the electrolyte due to their chemical inertness. An example of such behavior has been demonstrated in the addition of dichloromethane to a highly concentrated electrolyte where the co-solvent did not alter the interfacial properties of the electrolyte (i.e., electrochemical stability) but decreased the viscosity of the electrolyte.^27^ However, recent work casts doubt on the non-interacting nature of the co-solvent since it revealed that the addition of a highly fluorinated ether as a co-solvent to a concentrated electrolyte resulted in the change of the interfacial and bulk properties of the electrolyte concomitant with a change in the anion speciation in the electrolyte.^26^

The literature of pseudo-concentrated electrolytes also presents conflicting accounts of the role of the co-solvent on the ionic speciation and interfacial and bulk properties of the diluted electrolytes. In a work on a system composed of lithium bis(trifluoromethanesulfonyl)imide (LiTFSI) in ethyl acetate, the addition of a dichloromethane co-solvent did not appear to alter the speciation, which exists primarily as contact ion pairs (CIPs) and aggregates (AGGs) in diluted and pure electrolytes.^27^ In contrast, an electrolyte consisting of lithium bis(fluorosulfonyl)imide (LiFSI) in dimethyl carbonate (DMC) showed that the addition of a fluorinated co-solvent directly affects the speciation of the lithium ion by increasing the concentration of CIPs and AGGs in the diluted electrolyte.^28^ Owing to these conflicting reports, further study of the solvation structure of the lithium ion when diluted with a “non-interactive” co-solvent is needed.

**Scheme 1:**
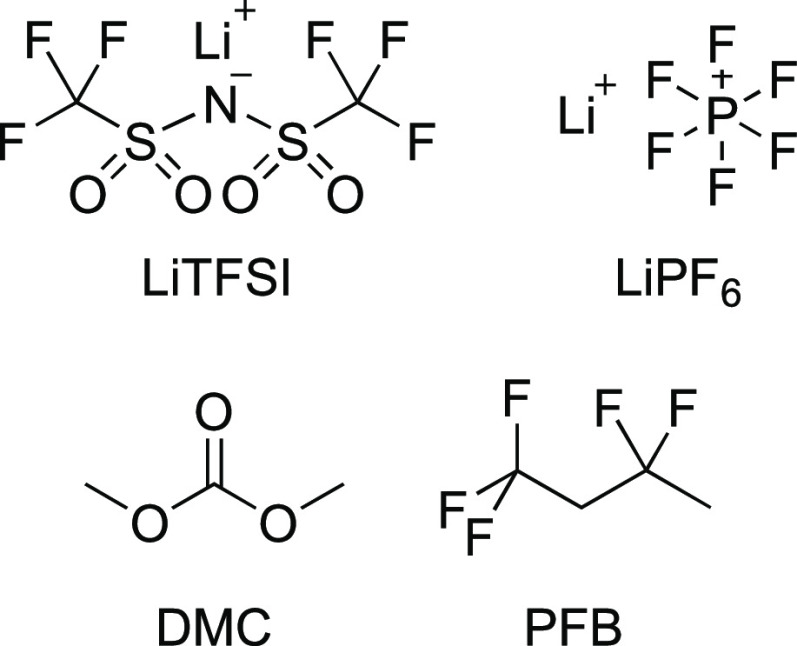
Chemical Structures of LiTFSI, LiPF_6_, DMC, and PFB

The focus of this study is to determine the possible molecular mechanisms by which a co-solvent affects the ionic speciation and the electrochemical properties of the system. Previous reports indicated that the chemical nature of the anion occupies a critical role in dictating speciation, so two different lithium salts are investigated to determine whether the identity of the anion plays any part in directing speciation.^29,30^ To this end, two commonly used lithium salts (lithium bis(trifluoromethanesulfonyl)imide, LiTFSI and lithium hexafluorophosphate, LiPF_6_) in DMC and a highly fluorinated solvent (1,1,1,3,3-pentafluorobutane, PFB) are used (Scheme 1). It has been demonstrated that both lithium salts are soluble in DMC, though not with the same degree of dissociation, and their use in previous spectroscopic studies of electrolytes warrants their use here as representative lithium electrolytes.^29−39^ The use of PFB as a co-solvent is based on the expectation that the solvent by itself will not affect the electrochemical window of the system due to its chemical inertness,^40,41^ but it will improve the transport properties because of its low viscosity.^42^ Moreover, PFB is structurally similar to the widely used polymer poly(vinylidene fluoride) typically used as a binder in lithium-ion batteries. Hence, electrolyte samples with varying co-solvent concentrations are examined experimentally via conductivity measurements, viscosity measurements, nuclear magnetic resonance (NMR) spectroscopy, linear FTIR spectroscopy, and cyclic voltammetry and complemented by density functional theory (DFT) computations. In particular, previous works have shown the benefits of using IR and NMR characterizations synergistically to study the solvation structure of lithium ions in a solution.^29,36−39,43,44^

## Methods

### Sample Preparation

Lithium hexafluorophosphate (LiPF_6_, 98% Acros Organics) was used as received. Lithium bis(trifluoromethanesulfonyl)imide (LiTFSI, 99%) was dried at 140 °C for 16 h under vacuum before use. Both lithium salts were stored in a N_2_-filled glovebox to avoid exposure to moisture. DMC (98% Acros Organics) and PFB (>99.5% Alfa Aesar) were dried under activated 4 Å molecular sieves to remove any trace amounts of water before use and stored in a N_2_-filled glovebox. Additionally, all solution preparation and sample cell assembly were conducted in the glovebox. The water content of these samples, tested via Karl Fischer titration, was determined to be 12 ppm. The solution concentrations in molarity and molality are included in Table S3 of the Supporting Information.

Sample cells assembled for FTIR of the carbonyl stretch region utilized a small volume of sample sandwiched between two CaF_2_ windows in an O-ring sealed sample cell without a spacer to overcome the known issue of high absorbance for the carbonyl stretch in DMC; this results in a path length of approximately 1–2 μm.

### FTIR Spectroscopy

FTIR spectra were recorded with a Bruker Tensor 27 spectrometer with a liquid nitrogen-cooled narrow band MCT detector. All samples were measured with a resolution of 0.5 cm^–1^ and were averaged from 40 scans at room temperature. Attenuated total reflectance FTIR (ATR–FTIR) spectra were recorded on a Bruker Tensor 27 spectrometer outfitted with a Pike Miracle ATR cell and a diamond/ZeSn crystal. The ATR–FTIR spectra were captured using a deuterated triglycine sulfate (DTGS) detector with a 4 cm^–1^ resolution, averaged over 16 scans.

### Conductivity and Viscosity Measurements

Conductivity and viscosity measurements were performed with a YSI 3200 conductivity meter and a Brookfield DV-II + Pro viscometer, respectively.

### NMR Spectroscopy

NMR spectroscopy was performed on a Bruker AVIII 500 MHz spectrometer. Shimming was performed with a chloroform reference. Hexafluorobenzene (C_6_F_6_) was used as the reference standard in all samples; sealed capillary tubes of C_6_F_6_ were inserted into each NMR tube to ensure that no direct interactions occur between the reference standard and the sample.

### Electrochemical Methods

Linear sweep voltammetry experiments were performed with an SP-300 Biologic potentiostat. The scans were conducted at a 100 mV/s scan rate. For all the samples, the working and counter electrodes were made of platinum, and Ag/Ag^+^ was used as a pseudo-reference electrode. For comparison to the Li/Li^+^ standard used in much electrochemical literature, scans were also conducted with the addition of a small amount of ferrocene (as an absolute internal standard) and referenced versus Li/Li^+^ using a conversion as outlined in the literature.^45,46^ The onset potentials were determined as the intersection potential of the tangent lines to the capacitive and faradaic currents.

### DFT Calculations

DFT calculations were performed in the Gaussian 09 software to complement experimental NMR data.^47^ Geometry optimizations and chemical shifts were computed using the PBE functional and the 6-311++G** basis set. The functional and basis set were chosen based on a previous demonstration that they correctly model the lithium solvation shell and their speciation in solution.^29,48−53^ NMR chemical shifts were calculated using the Gauge-independent atomic orbital method.^54^

## Results

**Figure 1:**
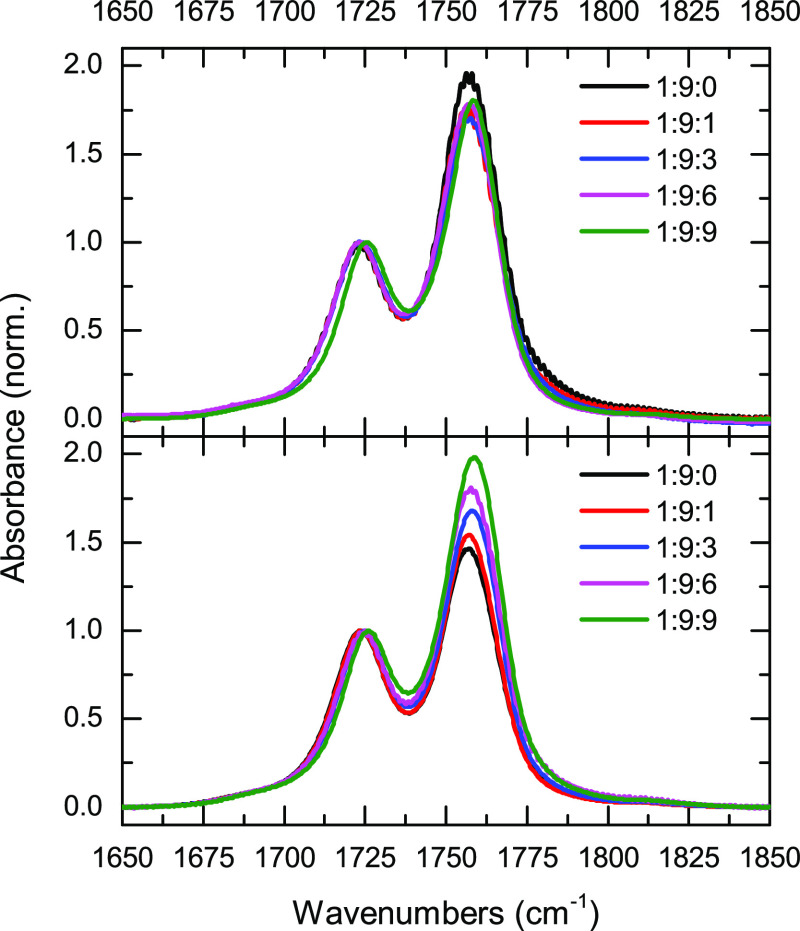
FTIR spectra of the carbonyl stretch region for LiTFSI/DMC/PFB samples (top) and LiPF_6_/DMC/PFB samples (bottom) as a function of PFB concentration.

**Figure 2:**
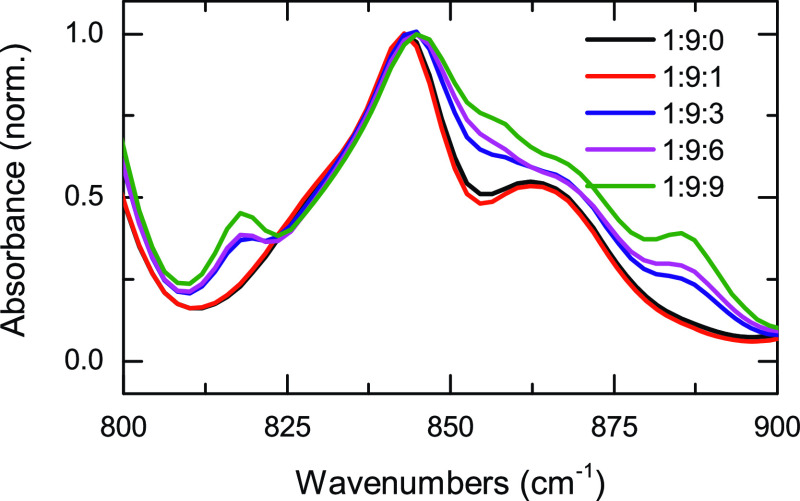
ATR–FTIR in the P–F stretch region for different LiPF_6_/DMC/PFB samples.

The FTIR spectra in the carbonyl stretch region (1650–1850 cm^–1^) for the samples containing either LiPF_6_ or LiTFSI in solvent mixtures with different molar ratios of DMC and PFB are shown in Figure 1. The spectra for both samples show two bands with a 2:1 intensity ratio separated by ∼35 cm^–1^. In LiTFSI samples, the high- and low-frequency bands are located at 1757 and 1724 cm^–1^, respectively. The initial addition of PFB slightly decreases the height ratio between the high- and low-frequency bands, but it remains almost equal when the concentration of PFB is further increased. Similar spectra are observed for the LiPF_6_ samples. However, the higher frequency band (1760 cm^–1^) increases its intensity with the addition of PFB. Moreover, the lower frequency band (1725 cm^–1^) shows a noticeable blue shift with increasing PFB concentration. Complementarily, Figure 2 shows the ATR-FTIR spectra in the P–F stretch region for the LiPF_6_ samples. There are five bands at 818, 844, 857, 863, and 885 cm^–1^ in these samples. While the bands at 844 and 863 cm^–1^ are present at all compositions, the bands at 818, 857, and 885 cm^–1^ are only visible at PFB concentrations of 1:9:3 LiPF_6_/DMC/PFB or greater. In addition, the peaks at 844 and 863 cm^–1^ blue-shift with increasing PFB content, while the other three bands appear to only grow with increasing PFB content.

**Figure 3:**
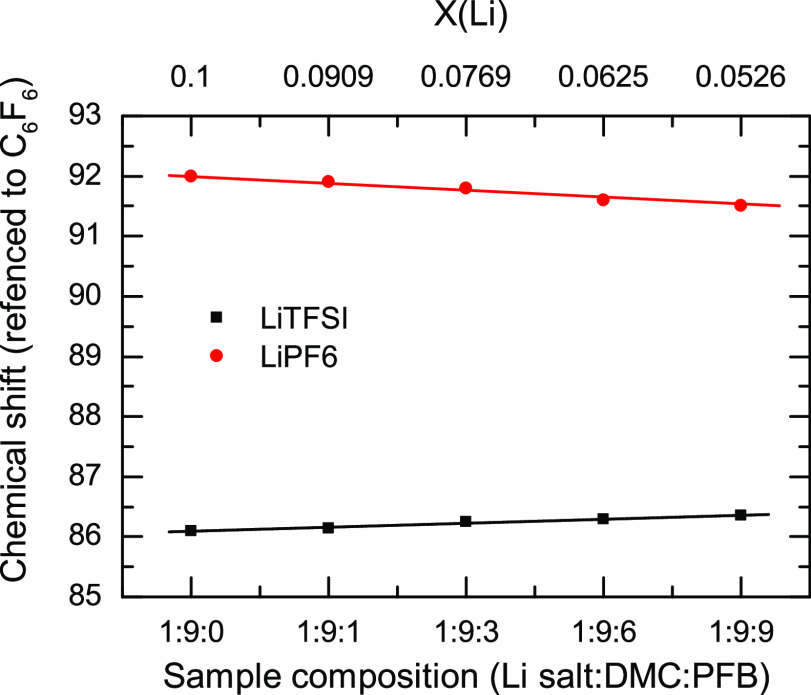
Chemical shift of the ^19^F nuclei as a function of sample composition (bottom axis) and molar fraction of lithium (*Χ*(Li), top axis) for both LiTFSI/DMC/PFB samples (black squares) and LiPF_6_/DMC/PFB samples (red circles).

NMR experiments were also performed to investigate the anion speciation using the ^19^F nuclei since TFSI^–^ does not have easily identifiable vibrational modes that change with speciation. The chemical shifts for both lithium salts in the different solvent mixtures are presented in Figure 3. The ^19^F nuclei in LiPF_6_ samples show a decrease of the chemical shift with increasing concentration. Conversely, the LiTFSI samples present an upshift in the chemical shift with increasing PFB.

**Figure 4:**
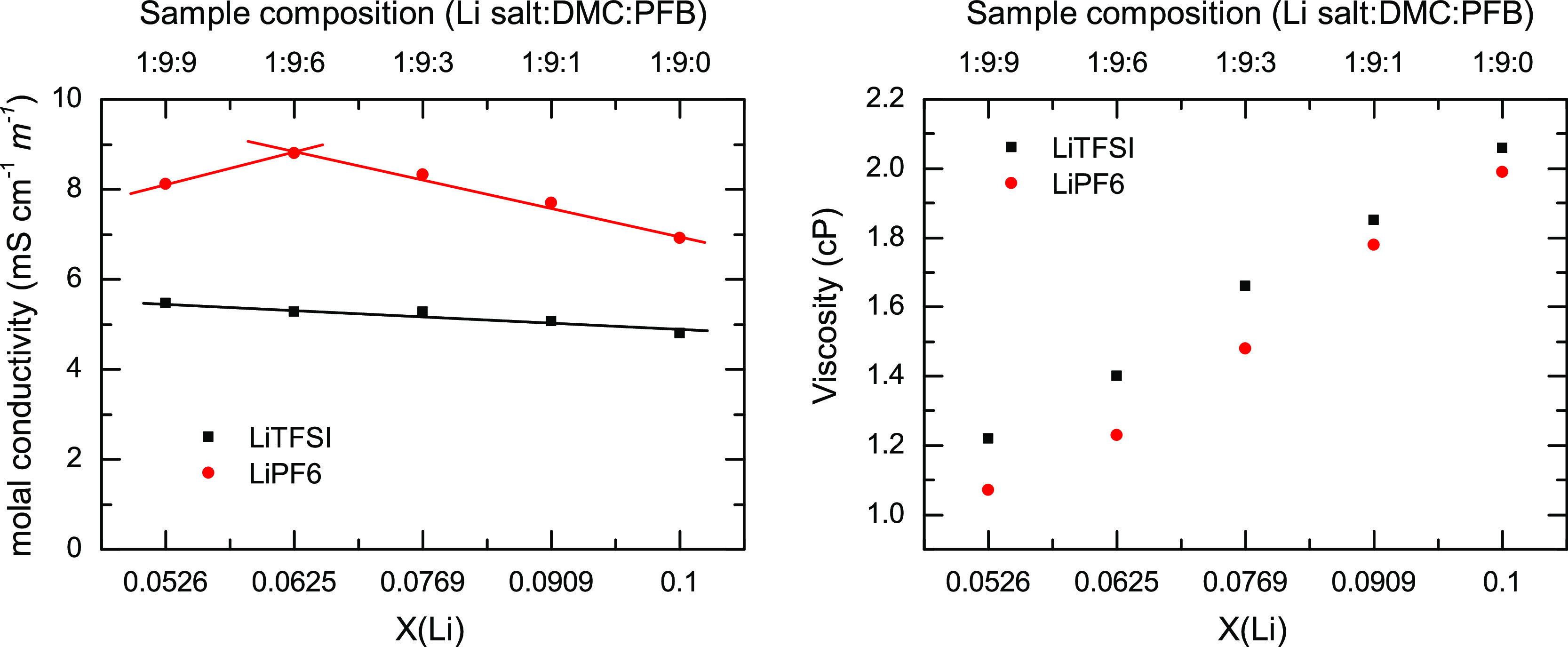
Left panel contains the molal conductivities of LiPF_6_/DMC/PFB (red circles) and LiTFSI/DMC/PFB (black squares) samples with respect to sample composition (top axis) and molar fraction of lithium (*Χ*(Li), bottom axis). The right panel depicts the viscosities for LiTFSI and LiPF_6_ samples with respect to sample composition (top axis) and molar fraction of lithium (*Χ*(Li), bottom axis). The conductivities were taken at room temperature, 25 °C. The temperature was held at 23 °C during viscosity measurements via a circulating chiller.

Additionally, the molal conductivity of LiPF_6_/DMC/PFB and LiTFSI/DMC/PFB samples at several concentrations at room temperature was also studied, and the results are presented in Figure 4 with respect to both PFB concentration and Li concentration. The conductivity of the LiTFSI/DMC/PFB samples is almost constant at ∼ 5 mS·cm^–1^·m^–1^, save for the 1:9:0 sample, which shows slightly lower conductivity. For the LiPF_6_/DMC/PFB samples, the conductivity generally shows a steady increase with PFB concentration. The viscosity measurements of all samples are shown in Figure 4. The viscosity decreases with PFB concentration for all compositions for both LiTFSI and LiPF_6_ samples.

**Figure 5:**
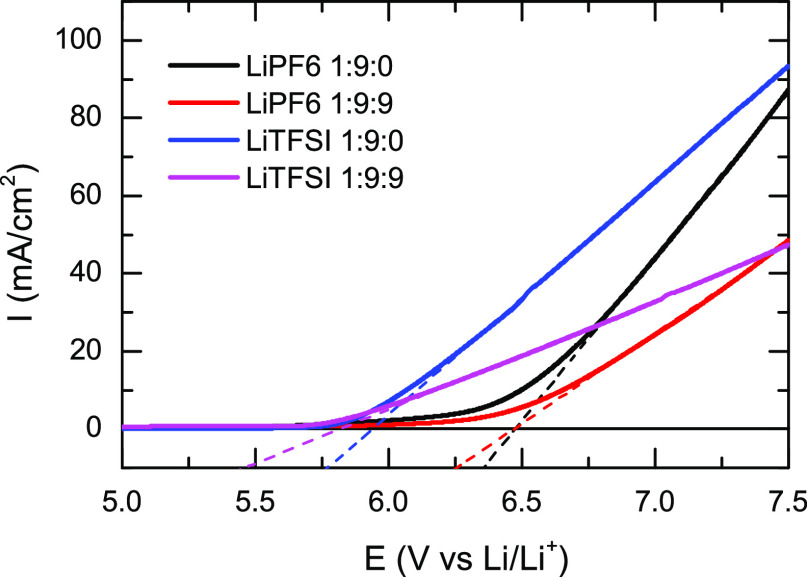
Linear sweep voltammogram for 1:9:0 and 1:9:9 compositions of the LiPF_6_/DMC/PFB (black and red lines, respectively) and LiTFSI/DMC/PFB (blue and magenta lines, respectively) samples. The intersection of the dashed lines with *I* = 0 mA/cm^2^ provides a qualitative difference of the onset potential. The change in current density over a varied potential range is plotted for the oxidative scan of the first trace for each composition at a 100 mV/s scan rate.

Finally, the electrochemical response of the samples was also studied via linear sweep voltammetry (Figure 5). For this study, two different limiting PFB concentrations (1:9:0 and 1:9:9) were studied for both LiPF_6_ and LiTFSI electrolytes. In the LiPF_6_ samples, the voltage ranges show that the onset of oxidation occurs at 6.48 V versus Li/Li^+^ for the 1:9:0 concentration and 6.47 V versus Li/Li^+^ for 1:9:9, while in the case of LiTFSI samples, oxidation occurs at 5.94 V versus Li/Li^+^ for the 1:9:0 sample and 5.82 V versus Li/Li^+^ for the 1:9:9 sample.

## Discussion

### Ionic Speciation

**Scheme 2:**
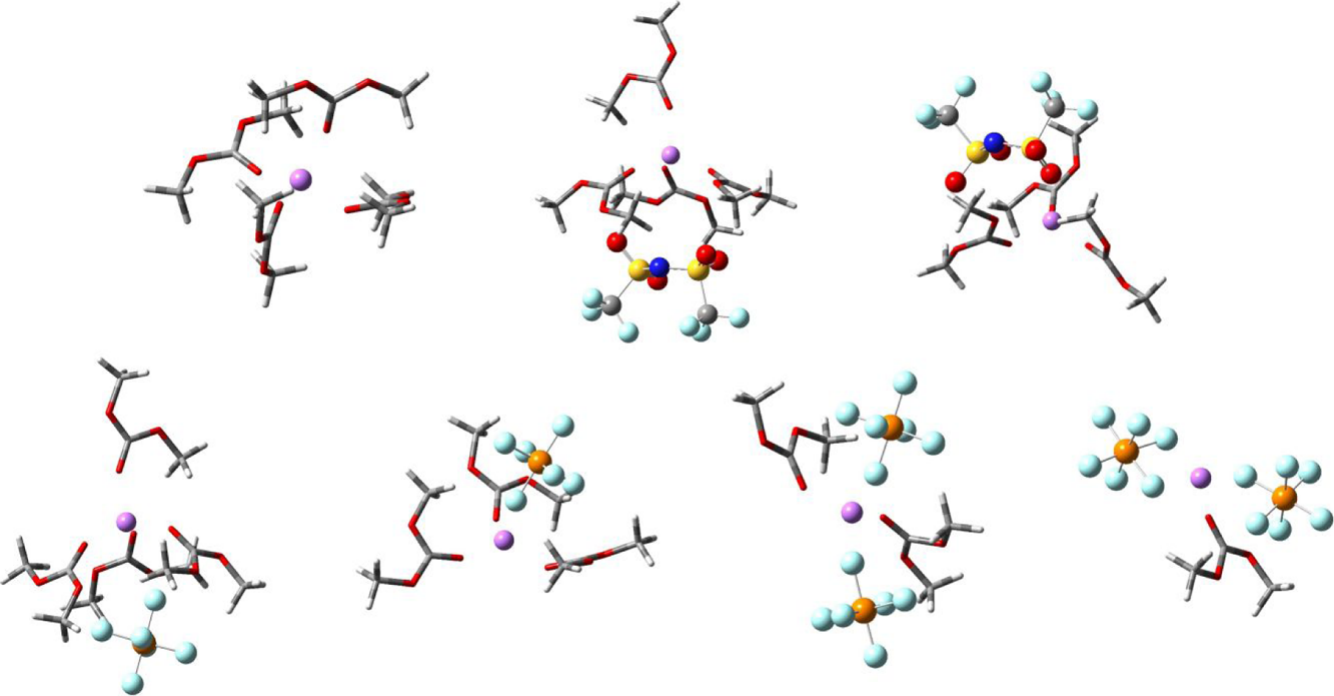
Possible Solvation Structures; Top Row, from Left to Right: Solvated Li^+^, LiTFSI SSIP, and LiTFSI CIP; Bottom Row, from Left to Right: LiPF_6_ SSIP, LiPF_6_ CIP, LiPF_6_ 2,2-AGG, and LiPF_6_ 1,2-AGG

The carbonyl stretching mode of the organic carbonate in the electrolytes has been previously used as a vibrational probe to study the solvation shell structure and ion speciation because of its sensitivity to local interactions.^55−59^ In our study, the carbonyl stretching mode of DMC was used as a vibrational probe to investigate changes in the local lithium ion environment produced by the addition of PFB concentration since the high- and low-frequency bands (1757 and 1724 cm^–1^) have been assigned to the free carbonyl- and lithium-coordinated carbonyl stretches, respectively.^38,60,61^ The linear FTIR spectra for LiTFSI and LiPF_6_ samples (Figure 1) show definitely different trends in association with the solvent with the lithium ion for the two salts. In the case of LiTFSI samples, the spectra reveal a downward trend where the amount of free carbonate is maximized for the sample without PFB, and the first addition of PFB slightly lowers the amount of free solvent, which then remains almost constant for all the different PFB concentrations. This trend implies that PFB is not likely to directly interact with Li^+^. However, it appears that the addition of PFB to the LiTFSI samples actually enhances the interaction of the organic carbonate with lithium ions. This is explained by the extremely poor solubility of LiTFSI in PFB, even though PFB has a larger dielectric constant than DMC.^62^ In regard to ion speciation, the changes in the carbonyl stretch band intensities indicate that in the absence of PFB (the 1:9:0 LiTFSI sample), some CIPs are present, but the addition of PFB (the 1:9:1 sample) is sufficient to disrupt such complexes, resulting in a higher coordination of lithium ions by carbonates, or equivalently, in the loss of intensity for the free carbonyl band (1757 cm^–1^). Moreover, subsequent additions of PFB do not appear to change the free carbonate solvent molecules or the ionic speciation in the sample since the ratio of the bands does not change. Note that the loss in the intensity of the free carbonyl band could also be attributed to a change in the dielectric constant of the solution by the addition of PFB, but this is unlikely because the ratio of the band intensity remains invariant even at very large concentrations of PFB. The solvation structures described are shown in Scheme 2.

The linear IR spectra of the LiPF_6_ samples (Figure 1) also show two bands in almost the same positions as the LiTFSI samples. Compared to the bands in the latter, the carbonyl stretch bands are blue-shifted by 1 and 3 cm^–1^, respectively. Interestingly, the trend observed for the peak intensity of the LiTFSI samples is completely different from that of the LiPF_6_ samples, where a sustained growth of the free carbonyl band is observed with increasing PFB concentration. The trend in the carbonyl bands shows that more free solvent molecules are generated with the increasing concentration of PFB. While the addition of PFB could significantly alter the oscillator strength of the carbonyl mode of DMC, this effect should also be noticeable in the LiPF_6_ samples. However, the PFB addition to the LiPF_6_ samples only produces a small solvatochromic shift of the carbonyl stretch frequency.^63^ Thus, changes in the environment do not appear to be caused by substantial change in the transition dipole magnitude of the carbonyl stretch. The effect of PFB can be explained by an increase in the interaction between the lithium ion and its counterion, which results in more CIPs and AGGs in the LiPF_6_ samples with increasing PFB concentration. The formation of more CIPs and AGGs is also supported by the broadening revealed in the high-frequency band with increasing PFB concentration, which may denote additional vibrational modes from CIPs or AGGs contributing to the high-frequency band.^35^ This is reflected in the IR frequencies calculated from DFT (Figure S1), which shows that the high-frequency carbonyl band observed for the free species overlaps with bands predicted for the SSIP, CIP, and 2,2-aggregate species (structures B, C, and D in Scheme S1; see the Supporting Information). Furthermore, previous experimental and computational studies on LiPF_6_ in DMC suggest that the solution contains primarily SSIPs, such that stronger Li–anion interactions would result in aggregate species.^33,35,64^

The change in the speciation of the PF_6_^–^ ion is further deduced from ATR–FTIR spectroscopy. The P–F stretches of the anion in LiPF_6_ in DMC/PFB samples (Figure 2) show that, in the absence of PFB (1:9:0 sample), the ATR–IR spectrum presents two broad bands, one at 840 cm^–1^ and a second one at 860 cm^–1^. This latter band has been previously linked to the presence of SSIPs.^29,33,38^ At higher concentrations of PFB (1:9:3 and higher), three additional bands appear at 820, 857, and 885 cm^–1^, suggesting a change in the ionic speciation of PF_6_^–^. The proposed change in speciation is in agreement with a previous report, which showed the appearance and growth of the same side P–F stretch bands at high concentrations of LiPF_6_ in organic carbonates, where the interaction between the lithium ion and its counterion is enhanced by concentration.^35^ Moreover, this previous study also assigned the same P–F stretch bands, observed for the 1:9:3 samples and beyond, to the formation of aggregates.^35^ Hence, the LiPF_6_/DMC/PFB samples show increased ionic interactions with higher PFB concentrations. This behavior is in agreement with the increase in free carbonate molecules seen in the carbonyl stretch band for the samples with high PFB concentrations.^26^ DFT frequency calculations for the different LiPF_6_ species validate our assignment since the P–F stretch region (Figure S2) has a better agreement for frequencies of the free anion and the SSIP than for those of the CIP. Furthermore, the calculations also reveal the IR bands seen in FTIR (Figure 1), and the three additional IR bands observed in ATR (Figure 2) spectra with increasing PFB concentration are better represented by the carbonyl and P–F stretches arising from the 2,2-aggregate rather than from the 1,2-aggregate (structures D and E in Scheme S1; see the Supporting Information). In summary, the change in the speciation of PF_6_^–^ is consistent with the behavior in pseudo-concentrated electrolytes, wherein the presence of a non-interacting co-solvent strengthens the cation–anion interactions.^26,28^

The anion speciation was also inferred from the fluorine nuclei chemical shifts. As can be seen in the IR spectroscopy data, the LiTFSI and LiPF_6_ samples present opposite trends for the change in the chemical shift (Figure 3). While the PF_6_^–^ fluorine nuclei decrease their chemical shift (increasing shielding) with PFB concentration, the TFSI fluorine atoms increase their chemical shift (less shielding). From the perspective of the sample concentration, an increase in the lithium concentration should lead to a decrease of the chemical shift (increasing shielding) due to the stronger interaction between the anion and the cation as previously demonstrated for a TFSI-based ionic liquid doped with LiTFSI.^65^ DFT calculations (Figure S3) validate the concentration effect in the NMR chemical shift since the formation of a CIP results in a decrease of the chemical shift (more shielded) of anion fluorine nuclei in either sample. Moreover, the addition of an explicit solvation shell for Li^+^ does not modify the chemical shift trend (Figure S3). Hence, the DFT results confirm our interpretation of the IR data for both PF_6_^–^ and TFSI^–^. In other words, the addition of PFB to the LiPF_6_ sample leads to a stronger lithium–anion interaction, resulting in a more shielding of the fluorine nuclei (lower chemical shift) and more free carbonates. In contrast, the addition of PFB to the LiTFSI sample results in a weaker interaction between the cation and TFSI^–^ or equivalently a higher chemical shift (less shielding) in agreement with the disappearance of free carbonates in the sample as can be seen from the IR results.

So far, two key conclusions can be extracted from the experimental IR and NMR data and the computational results. First, the addition of PFB appears to affect the Li–anion interaction differently for the two samples. Second, the presence of the co-solvent has a stronger effect on the formation of CIPs and AGGs than lowering the lithium concentration. Hence, the addition of the co-solvent affects the speciation of the two electrolytes differently, which points to different molecular interactions between PFB and either anion. DFT calculations (see Table S1 in the Supporting Information) revealed that the interaction between the most energetically favored conformation of TFSI^–^ and PFB is favored by ∼3.6 kJ/mol over the interaction between PF_6_^–^ and PFB (see Scheme S3 in the Supporting Information). Previous work has shown the existence of weak hydrogen bonds between C–H donors and the O atom of acceptors.^66−70^ These CH···O hydrogen bonds have energetics on the order of 1 kcal/mol, different C–H bond lengths as compared to non-forming hydrogen-bonded species, and nearly linear CH···O geometries.^66,67,71−73^ The PFB–TFSI interaction shows similar energetics of ∼0.86 kcal/mol to the CH···O hydrogen bond. Additionally, the bond lengths for the C–H bonds of PFB and C–H–O bond angles (Table S2) mirror results previously seen in other systems (see refs (71) and (67), respectively). Thus, the DFT results strongly suggest that the formation of weak hydrogen bonds between PFB and the TFSI^–^ might be the cause for the dissociation of the CIPs in the LiTFSI/DMC samples. Overall, the juxtaposing results from IR, NMR, and DFT data provide a unified picture of the molecular–level interactions in these systems. The IR experiments show more free solvent molecules with increasing co-solvent concentration for the LiPF_6_/DMC system due to enhanced interactions between PF_6_^–^ and the lithium cation. Conversely, for the LiTFSI/DMC system, there is a slight loss of free solvent molecules with the initial introduction of PFB, indicating that the co-solvent dissociates ion pairs (SSIPs and CIPs) and forces the carbonate solvent to solvate Li^+^. In the case of the PF_6_^–^ sample, the enhanced cation–anion interactions are also observed through both the downshift of the fluorine chemical shift and the appearance of new bands in the P–F stretch IR region. In contrast, the weakening of the Li^+^–anion interaction produced by the presence of the co-solvent in the LiTFSI/DMC sample is directly observed from the upshift of the fluorine chemical shift. The NMR and IR spectra for these samples calculated with DFT strengthen the interpretation of these interactions, denoting the existence of free TFSI^–^ and SSIPs in the LiTFSI samples and free PF_6_^–^, SSIP, and 2,2-aggregate species in the LiPF_6_ samples.

### Macroscopic Properties

The electrophysicochemical properties of these systems also present interesting trends. The molal conductivity of both systems (Figure 4) shows that the value remains nearly constant with a slight increase for those samples containing PFB for the LiTFSI samples. In contrast, the LiPF_6_ samples present a maximum around the 1:9:6 sample, but all the samples containing PFB have a larger conductivity than the pure electrolyte (1:9:0 sample). In these electrolytes, the conductivity mechanism is expected to be driven by the mobility of free ions, the viscosity, and the lithium salt concentration.^74^ The formation of CIPs and AGGs should limit the number of charge carriers and thereby lower the conductivity of the system. However, the addition of a co-solvent to an electrolyte not only changes the number of charged species but also alters the viscosity of the system. Thus, a lower viscosity in the sample should facilitate a faster diffusion of the charge carriers, which is the observed behavior for pseudo-concentrated electrolytes.^19,28^

**Figure 6:**
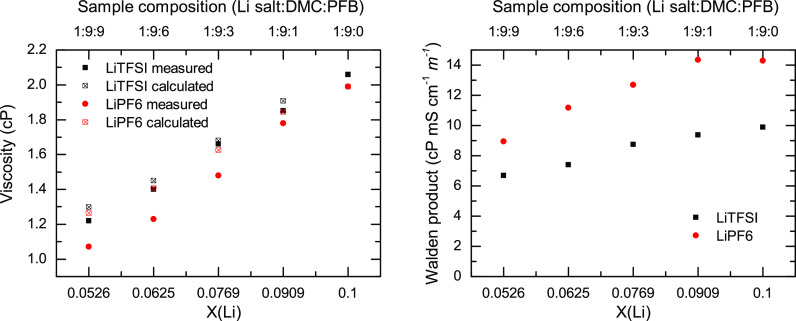
Left panel depicts the measured and calculated viscosities for LiTFSI/DMC/PFB (black full and crossed squares) and LiPF_6_/DMC/PFB (red full and crossed circles) samples. Right panel shows the Walden product for LiTFSI and LiPF_6_ samples at all compositions.

The viscosity of the studied electrolytes as a function of PFB concentration (Figure 4) in either sample displays a monotonic decrease of the viscosity with the addition of the co-solvent. Since viscosity and conductivity are inversely related, a monotonic decrease in viscosity should be reflected as an increase in the conductivity when the ionic speciation in the sample remains unaltered. This is the case for the LiTFSI samples, where the addition of PFB leads to a linear increase in conductivity. However, the trend does not explain the conductivity of the LiPF_6_ samples, which exhibits a nonlinear behavior (Figure 4), likely due to a change in the ionic speciation as previously concluded.^75^ Moreover, the viscosity of the LiPF_6_ samples does not follow an ideal behavior, in which the viscosity of the system is defined exclusively by the viscosity of the components and their molar fractions. The non-ideal behavior is seen when comparing the real and ideal viscosity of the mixtures (Figure 6), in which the latter was computed using the viscosity of the pure electrolytes and pure PFB.^41^ In this case, it is observed that the experimental viscosity for the LiTFSI samples matches well with the value of the viscosity predicted for the ideal case. In contrast, the experimental viscosity for the LiPF_6_ samples presents a much lower value than that of the ideal case. Thus, the viscosity provides evidence of a change in the cohesive forces of the electrolyte likely arising from the change in the ionic speciation of the LiPF_6_ sample due to the presence of PFB.

The effect of dilution in the samples on the ion transport is better observed using the viscosity-weighted conductivity (Walden product, Figure 6) as a function of PFB concentration. Because the Walden product removes the viscosity effect, one can use this metric to evaluate the changes in the number of charge carriers.^76^ For the LiTFSI sample, the Walden product (Figure 6) decreases monotonically since it is directly influenced by the change in the number of charge carriers produced by the addition of the co-solvent, that is, dilution. Contrarily, the Walden product (Figure 6) for the LiPF_6_ samples does not follow the same trend as LiTFSI, indicating that there is another underlying molecular mechanism, beyond simple dilution, altering the properties of the system. This mechanism is the change in ionic speciation of the two systems as derived from the IR and NMR experiments. Hence, the Walden products assert that PFB plays a different role in the solvation of these LiPF_6_ and LiTFSI electrolytes and their dilutions. In the case of the LiTFSI system, PFB has a minimal effect on the conductivity of the system and only serves as a co-solvent, while in the LiPF_6_ system, PFB creates a pseudo-concentrated electrolyte with enhanced conductivity by altering the ionic speciation of the electrolytes, that is, creating more aggregates.

Finally, the effect of PFB on the electrochemical stability of the electrolyte was also evaluated from cyclic voltammetry. It has been previously shown that there is a relationship between enhanced ionic conductivity and a change in the electrochemical stability of the electrolytes, in which a higher concentration of the lithium salt typically leads to a larger electrochemical stability.^10,17,18^ The linear sweep voltammogram (Figure 5) for the LiPF_6_ sample without (1:9:0) and with (1:9:9) PFB shows that the onset potential for oxidation is 6.48 and 6.47 V for the two samples, respectively. The results indicate a negligible (∼10 mV) change in the electrochemical window upon addition of PFB for the LiPF_6_ electrolyte. The LiTFSI samples show a completely different behavior, where the onset oxidation potential is found to be 5.94 and 5.82 V for the pure (1:9:0) and diluted (1:9:9) sample, respectively. Thus, in the LiTFSI electrolyte, the electrochemical stability window is reduced by ∼120 mV when PFB is added. Addition of PFB lowers the oxidative current densities in both electrolytes; however, the effect of PFB addition on the onset potentials differs depending on the lithium salt in the electrolyte. The electrochemical results appear to be conflicting, but the change in the bulk ionic speciation of the samples explains the observed electrochemical behavior since it has been previously observed that an increase in the cation–anion interaction leads to a larger electrochemical window of the electrolyte.^10,77^

To find a possible link and rationalize the effect of the bulk ionic speciation on the observed electrochemical behavior, the HOMO–LUMO gap of the different ionic species deduced from the experiments is calculated using DFT computations (see Methods section). This method is used because it has been previously shown that the HOMO–LUMO gap is correlated with the electrochemical stability.^78−80^ The HOMO–LUMO gap for the free anion, SSIP, and CIP of both salts, as well as aggregate species, is shown in Table 1. In the species related to LiTFSI, the HOMO–LUMO gap is found to be similar for the CIP and SSIP but significantly smaller for free TFSI^–^. A similar trend is seen for the LiPF_6_ species (free PF_6_^–^ < 1,2-AGG < 2,2-AGG ≈ SSIP ≈ CIP), albeit the consideration of aggregates as previously demonstrated. Thus, the electrochemical data allow us to deduce the same molecular mechanism resulting from the addition of PFB to the two electrolytes. In the case of the LiTFSI sample, a weaker interaction between Li^+^ and its counterion resulting in less ion pairs (both SSIPs and CIPs) and more free ions is observed when PFB is added. These changes in speciation cause a decrease in the electrochemical window. In contrast, the addition of the co-solvent leads to a stronger Li–anion interaction in the LiPF_6_ electrolyte, which leads to the formation of more CIP and AGGs in the sample and maintains the electrochemical window constant. Overall, the contrasting behaviors observed in the electrochemical stability of the samples reflect a shift in speciation toward more free ions (with reduced electrochemical stability) in the case of the LiTFSI electrolytes and a shift toward aggregates (with a similar electrochemical stability) in the case of LiPF_6_ electrolytes.

**Table 1:** HOMO–LUMO Gap, Computed via DFT, for Different Species of LiTFSI and LiPF_6_

species	HOMO–LUMO gap (eV)
free TFSI^–^	2.51
LiTFSI SSIP	7.23
LiTFSI CIP	7.51
free PF_6_^–^	3.92
LiPF_6_ SSIP	8.10
LiPF_6_ CIP	8.57
LiPF_6_ 2,2-AGG	8.05
LiPF_6_ 1,2-AGG	4.98

## Summary

Two non-aqueous lithium ion electrolytes and their dilutions with a co-solvent were characterized using experimental and computational methods. Complementary IR and NMR spectroscopies showed a different speciation for the two electrolytes with the increasing concentration of the co-solvent. In the case of the LiPF_6_ electrolyte, the anion forms aggregates when PFB is added, while the addition of the co-solvent actually dissociates CIPs in the LiTFSI electrolyte. The change in the speciation is also observed in the different electrophysicochemical properties of the systems. The stronger Li–anion interactions in the LiPF_6_ electrolyte result in a higher conductivity, lower viscosity, and no change in the electrochemical window with the addition of the co-solvent. In contrast, the conductivity of the LiTFSI electrolyte remains fairly constant irrespective of the PFB concentration, while the addition of the co-solvent decreases both the viscosity and the electrochemical window. The lower oxidative stability is in agreement with the change in the speciation and their computed electrochemical stability via the HOMO–LUMO gap. The difference in speciation appears to be caused by the formation of a strong hydrogen bond between TFSI^–^ and the co-solvent. In short, this study establishes that the addition of a co-solvent, usually considered inert, can alter the ionic speciation of the electrolyte species, and attempts to improve the transport properties through a decrease in viscosity could inadvertently impact the electrochemical properties of the system.

## Supporting Information Available

All species investigated via DFT computations; calculated IR frequencies for the carbonyl stretch of the solvent as a function of ionic speciation; calculated IR frequencies in the P–F stretching for SSIPs, CIP, and AGGs; calculated NMR chemical shift for different ionic species; and energetics for the interaction between PFB and each anion (PDF)
