# Supplementary material for: Tale of a “Non-interacting” Additive in a Lithium-Ion Electrolyte: Effect on Ionic Speciation and Electrochemical Properties
Source: J Phys Chem C Nanomater Interfaces. 2022 Jan 24;126(4):2141–50. doi: 10.1021/acs.jpcc.1c09193 (PMC8820140; doi:10.1021/acs.jpcc.1c09193)
Supplement: Supplementary file 1 — jp1c09193_si_001.pdf [file jp1c09193_si_001.pdf]

Electronic supplementary information

The Tale of a “Non-interacting” Additive in a Lithium Ion Electrolyte: Effect on Ionic Speciation and Electrochemical Properties

*Jeramie C. Rushing, Callie M. Stern, Noemi Elgrishi and Daniel G. Kuroda**

Department of Chemistry, Louisiana State University, Baton Rouge, Louisiana 70803, United States

*Address correspondence to dkuroda@lsu.edu

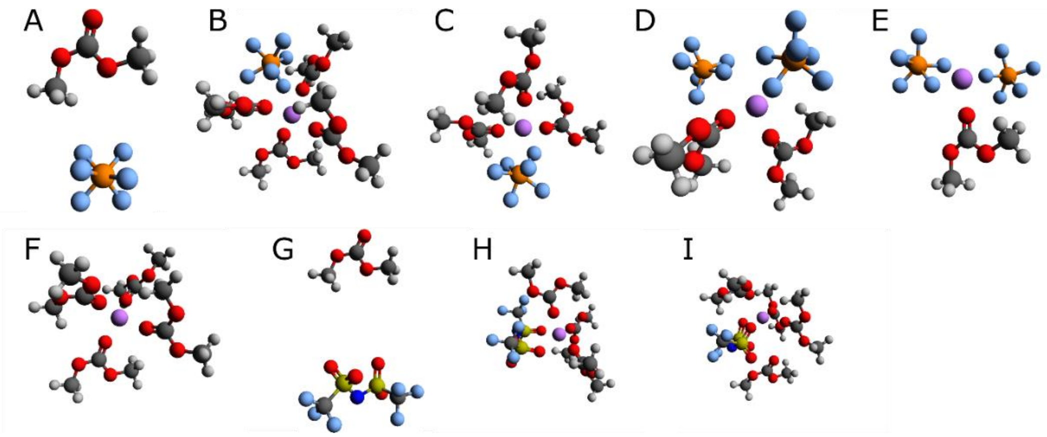

Scheme S1. All species investigate via DFT computations: Free PF_6^- anion (A), $LiPF_6$ SSIP (B), $LiPF_6$ CIP (C), $LiPF_6$ 2,2-AGG (D), $LiPF_6$ 1,2-AGG (E), solvated Li^+ cation (F), free TFSI- anion (G), LiTSFI CIP (H), and LiTFSI SSIP (I).

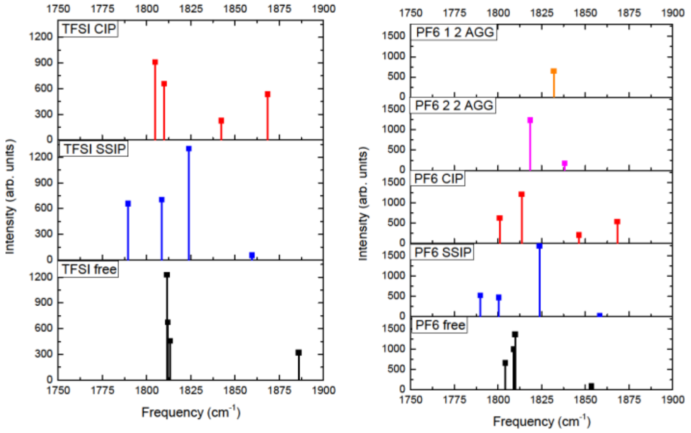

Figure S1. Calculated IR frequencies for the carbonyl stretch of the solvent for $LiPF_6$ and LiTFSI species without addition of PFB (all species are shown in Scheme S1).

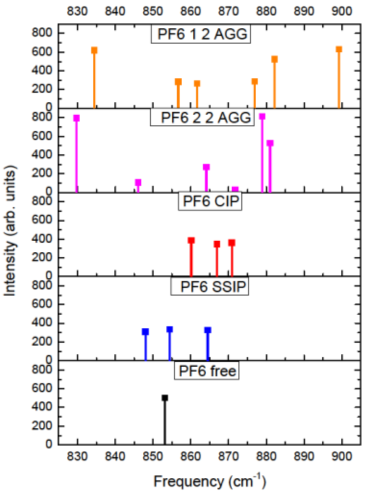

Figure S2. Calculated IR frequencies in the P-F stretching region for different $LiPF_6$ speciation (Scheme S1).

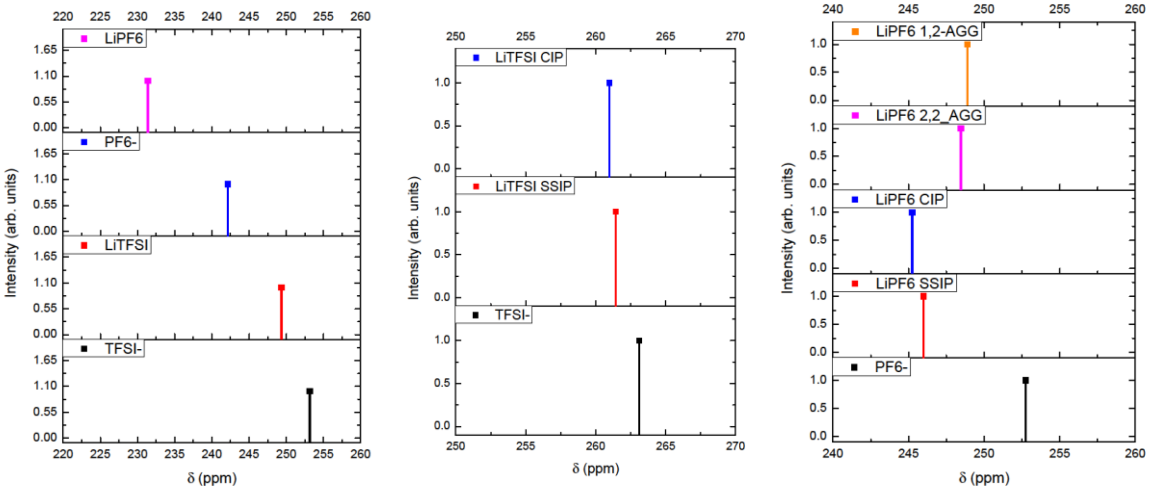

Figure S3. Calculated NMR shielding for the addition of Li^+ to PF_6^- and TFSI- anions. Left panel without solvent, and middle and right panels with explicit solvent (as shown in Scheme S1).

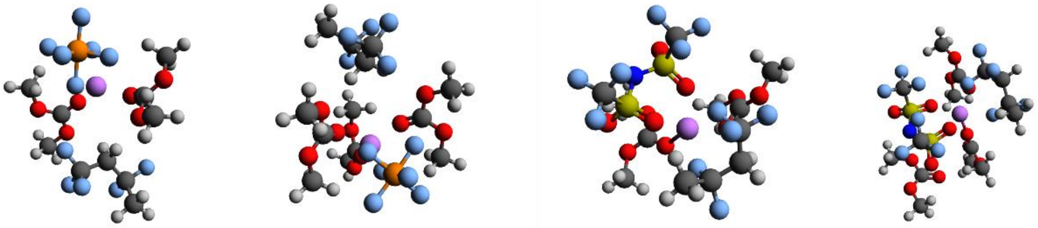

Scheme S2. Contact ion pair with the explicit carbonates and PFB in the solvation shell. From left to right: $LiPF_6$ CIP, $LiPF_6$ SSIP, LiTFSI CIP, LiTFSI SSIP.

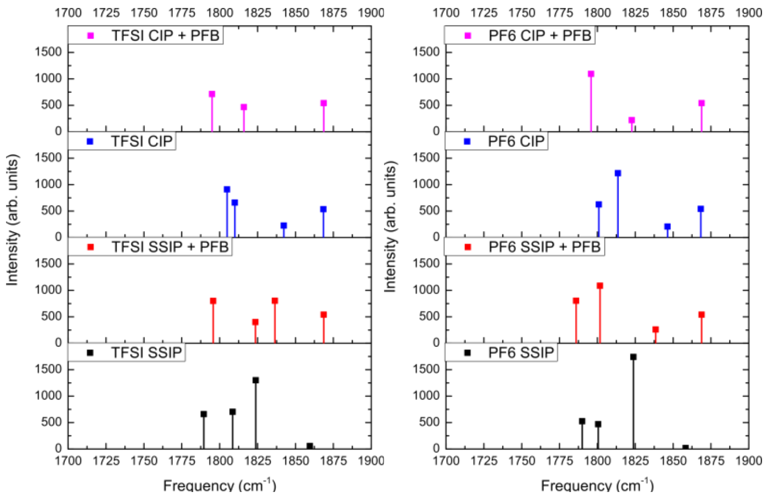

Figure S4. Calculated IR frequencies for carbonyl region of LiTFSI (left) and $LiPF_6$ (right) ion pair species with the addition of PFB (Scheme S2). The frequencies for the SSIP and CIP species without PFB are included for comparison.

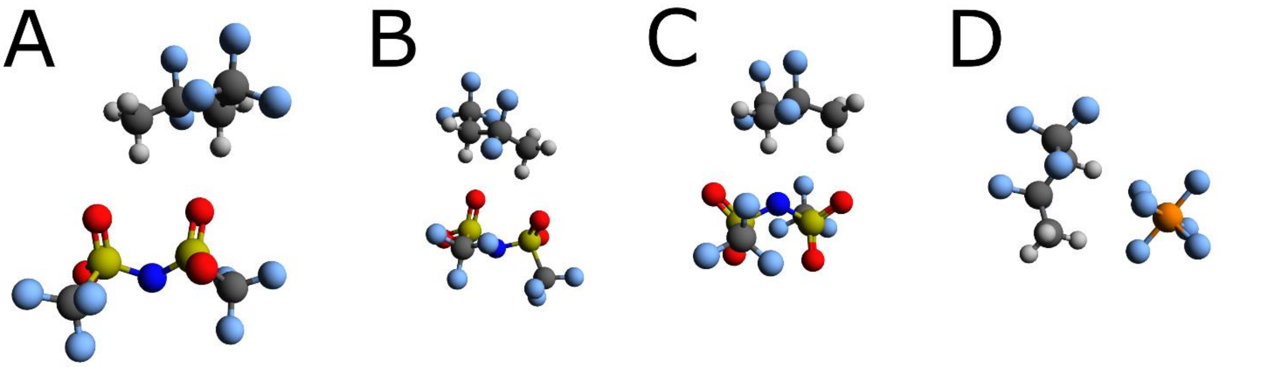

Scheme S3. The atomistic structures used to investigate the hydrogen bond energetics for each anion (Table S1).

Table S1. Energetics for the interaction between PFB and each anion (Scheme S3).

Interaction	ΔE (vs cis-cis TFSI-)	ΔE (vs cis-trans TFSI-)	ΔE (vs PF_6^-)
(A)PFB(H) --- ccTFSI(O)	-51.43 kJ/mol	-48.05 kJ/mol	-
(B) PFB(H) --- ccTFSI(O)	-53.84 kJ/mol	-50.45 kJ/mol	-
(C) PFB(H) --- ctTFSI(O and N)	-59.02 kJ/mol	-55.64 kJ/mol	-
(D) PFB(H) --- PF_6(F)	-	-	-55.40 kJ/mol

Table S2. C-H bond lengths and bond angles (C-H-O and C-H-N) for the most favorable TFSI-PFB interaction (C in Scheme S3). C-H bond lengths in non-interacting TFSI- are included for comparison.

Sample	C-H bond length (CH_2)	C-H bond length (CH_3)	C-H-O bond angle	C-H-N bond angle
TFSI-	1.0921 Å	1.0908 Å	---	---
TFSI-PFB	1.0955 Å	1.0922 Å	165°	170°

Table S3. Molality of all samples tested.

	Molality				
Sample	1:9:0	1:9:1	1:9:3	1:9:6	1:9:9
LiTFSI	0.91m	0.80m	0.65m	0.50m	0.41m
LiPF6	1.04m	0.90m	0.71m	0.54m	0.44m
